# Venous Excess Ultrasound Score and Acute Kidney Injury in Patients With Acute Heart Failure

**DOI:** 10.1016/j.jacadv.2026.102752

**Published:** 2026-05-27

**Authors:** Diego Araiza-Garaygordobil, Eder Gonzalez-Macedo, Eduardo Argaiz, Alejandro Ezquerra-Osorio, Luis Soliz-Uriona, Rodrigo Gopar-Nieto, Jose L. Briseño-De la Cruz, Manuel A. Candia-Ramirez, Jorge I. García-Espinoza, Rafael W. Sanez-Reyes, Salvador Lopez-Gil, Alejandro Sierra-Gonzalez De Cossio, Ana C. Maldonado-May, Ximena Latapi-Ruiz Esparza, Braiana A. Diaz-Herrera, Sarai Hernandez-Pastrana, Jorge A. Ortega-Hernandez, Edith Adame-Avilés, Alexandra Arias-Mendoza, Daniel Sierra-Lara Martinez

**Affiliations:** a Coronary Care Unit, Instituto Nacional de Cardiología Ignacio Chávez, Mexico City, México; b División de Estudios de Posgrado, Faculty of Medicine, National Autonomous University of Mexico, Mexico City, Mexico; c Tecnológico de Monterrey, Escuela de Medicina y Ciencias de la Salud, México; d Nephrology Department, Instituto Nacional de Cardiología Ignacio Chávez, Mexico City, México; e Departamento de Nefrología y Metabolismo Mineral, Instituto Nacional de Ciencias Médicas y Nutrición Salvador Zubirán, Mexico City, Mexico; f Plan of Combined Studies in Medicine (MD/PhD-PECEM), Faculty of Medicine, National Autonomous University of Mexico, Mexico City, Mexico

**Keywords:** acute heart failure, acute kidney injury, central venous pressure, VExUS

## Abstract

**Background:**

Systemic venous congestion is associated with an increased risk of acute kidney injury (AKI) in critically ill patients. Venous Excess Ultrasound Score (VExUS) has been proposed as a noninvasive tool for assessing venous congestion based on ultrasonographic evaluation of the inferior vena cava, hepatic vein flow, portal vein pulsatility, and intrarenal Doppler flow.

**Objectives:**

The authors aimed to evaluate the association between the VExUS score and AKI in patients with acute heart failure (AHF).

**Methods:**

Prospective study including patients with AHF (acute de novo and decompensated chronic heart failure). VExUS was performed within the first 8 hours of hospital stay. Patients were classified by presence of severe congestion (VExUS 0-1/2-3). The primary objective was AKI, defined by Kidney Disease: Improving Global Outcomes criteria. Secondary objectives included the composite of AKI, cardiogenic shock, and all-cause mortality.

**Results:**

A total of 109 patients were included. After ultrasound assessment, 41 (37.2%) were categorized as having significant congestion (VExUS >1). VExUS >1 was more frequent in chronic HF and in patients with right ventricular involvement. At each increasing VExUS grade, a higher proportion developed AKI: VExUS = 0 (29.2%), VExUS = 1 (44%), VExUS = 2 (66.6%), and VExUS = 3 (60.7%) (*P* = 0.008). A significantly increased risk of AKI (HR: 2.65; 95% CI: 1.39-5.07; *P* = 0.003) was found in patients with VExUS >1. Adjusted logistic regression showed VExUS >1 as the strongest predictor (OR: 4.60; 95% CI: 1.47-16.0) of AKI during hospital stay.

**Conclusions:**

In AHF patients, the VExUS score is associated with AKI occurrence. Further studies are needed to clarify the role of VExUS assessment in HF.

Acute heart failure (AHF) represents the leading cause of hospitalization in patients over 60 years old and it is frequently associated with acute kidney injury (AKI).^1^ In patients with AHF, the incidence of AKI is ∼20%, and AKI is recognized to be a strong independent predictor of both in-hospital and 1-year mortality.^2^

In the setting of AHF, AKI can occur from hemodynamic and neurohormonal activation, venous congestion, and nephrotoxic medications.^3^ A pivotal study in 2018 showed that elevated central venous pressure is the main hemodynamic factor driving kidney dysfunction in patients with acute decompensated heart failure.^4^ Venous Excess Ultrasound Score (VExUS) has been proposed as a noninvasive score to assess systemic venous congestion.^5^ Recently, a prospective study including patients hospitalized with acute coronary syndrome showed that VExUS is a strong predictor for the occurrence of AKI.^6^ However, the association between VExUS and AKI in patients with AHF remains unclear.

The objective of the present study was to evaluate the association between venous congestion assessed with VExUS and the incidence of AKI and a composite of adverse clinical outcomes (AKI, cardiogenic shock, and all-cause mortality) in patients with AHF.

## Materials and methods

### Study design

We conducted a longitudinal, prospective, single-center study including adult patients admitted to the emergency room between July 2023 and January 2024 with the diagnosis of AHF, and in whom VExUS evaluation was performed during the first 8 hours of hospital stay. No external funding was used to support this work. The protocol received local research and ethics committee approval and complies with the principles of the Declaration of Helsinki, with approval number PT-18-078. Written informed consent was obtained from all patients before enrollment in the study.

### Study population

Study patients included those aged 18 to 99 years old and consecutively admitted to the study center with the diagnosis of AHF for a hospital stay longer than 24 hours. Inclusion criteria mandated that VExUS was performed during the first 8 hours of hospital stay (either at the emergency department, intensive care unit, or hospital guards). Patients suffering an acute coronary syndrome (either ST-segment elevation myocardial infarction or non–ST-segment elevation myocardial infarction) as a trigger for their acute HF event were included, as long as a history of HF or pre-existing left ventricular ejection fraction (LVEF) <40% in the 12 months preceding the index event was documented. Patients with a previous diagnosis of end-stage kidney disease (Kidney Disease: Improving Global Outcomes [KDIGO] V) and with a previously estimated estimated Glomerular Filtration Rate (eGFR) of <15 mL, as well as those with conditions interfering with portal Doppler assessment (cirrhosis or portal thrombosis) were excluded from the study. Patients in whom VExUS exam was not feasible during the first 8 hours or those with poor acoustic windows (making echocardiographic evaluation impossible) were also excluded.

AHF was diagnosed and classified according to international nomenclature and clinical practice guidelines.^7^ AHF (either de novo HF or chronic decompensated HF) was defined as the new onset or worsening of symptoms (including dyspnea, decreased exercise capacity, fatigue, or other hypoperfusion or volume overload symptoms), physical examination findings compatible with HF (peripheral edema, pulmonary congestion, increased jugular venous pressure, or third heart sound), and complementary findings considered to be due to heart failure (increased levels of N-terminal pro–b-type natriuretic peptide [NT-proBNP] >1,800 pg/mL, radiographic evidence of pulmonary congestion and/or invasive or noninvasive evidence of increased filling pressures or decreased cardiac output).

### Data acquisition and VExUS assessment

Demographic and baseline clinical data, including relevant medical history, were collected prospectively by the study investigators. Blood and urine samples were obtained at admission to measure creatinine, NT-proBNP, and other relevant measurements. Admission eGFR was calculated using chronic kidney disease epidemiology collaboration (CKD-EPI) equation using initial serum creatinine levels.

The VExUS assessment is routinely performed in our emergency department. To ensure consistency and accuracy, all VExUS examinations were performed by a dedicated group of 3 cardiologists with formal previous training in Doppler echocardiography and point-of-care ultrasound. Prior to study initiation, operators underwent structured training for this purpose following the methodology described by Gómez-Rodríguez César et al (2023), which included supervised image acquisition, standardized waveform interpretation, and interobserver agreement assessment in routine clinical practice. This training has demonstrated excellent reproducibility, with an intraclass correlation coefficient of up to 0.94 and a Kappa statistic of up to 0.88.^8^ Based on this experience, VExUS acquisition and interpretation were standardized across operators throughout the study period. All measurements were performed using the same predefined acquisition protocol and interpretation thresholds.

For the assessment of VExUS, patients were positioned in a dorsal decubitus position and using standard 18 cm in-depth transducers to perform hepatic vein, portal vein, intrarenal venous Doppler, as well as inferior vena cava (IVC). The IVC diameter was measured in its intrahepatic portion of the junction with the hepatic veins using a longitudinal view from a subxiphoid position, the maximal IVC diameter (IVCDmax) and minimal IVC diameter (IVCDmin) were measured and the IVC collapsibility index with passive respiration was calculated using the formula *([IVCDmax x IVCDmin]/IVCDmax) x 100.*

Hepatic vein pulsatility was assessed by placing the probe in either a subxiphoid or lateral view and placing the Doppler gate across any of the hepatic veins and observing Doppler waveforms. Normal hepatic vein Doppler waveforms show a small, retrograde a-wave, followed by anterograde S and D waves, with the ratio of amplitudes of the S to D waves being >1. In increasing states of congestion, the S wave shrinks relative to the D wave before reversing entirely, becoming retrograde. An S:D ratio >1 was considered normal, an S:D ratio ≤1 mildly abnormal, and a reversal of the S wave severely abnormal. Hepatic portal venous pulsatility was assessed similarly, by placing the Doppler gate across the portal vein and observing the pulsatility index: *(Vmax – Vmin)/Vmax*. A normal portal vein Doppler waveform was considered with a pulsatility index <30%. A pulsatility index of 30% to 49% was mildly abnormal, and a pulsatility index >50% was severely abnormal.

Renal vasculature was visualized with the probe in the posterior axillary line, with the Doppler gate placed to detect the flow of the interlobar or arcuate renal veins in the renal cortex, outside the hilum of the kidney. A normal renal Doppler pattern shows arterial pulsations generating regular retrograde peaks, and renal veins generating a continuous, smooth anterograde flow. As venous congestion increases, venous pulsations become visible, creating anterograde pulsations observable during systole and diastole, and eventually, only diastole. A smooth venous baseline was considered normal. Biphasic anterograde pulsations reflecting systole and diastole were considered mildly abnormal, and monophasic pulsation, corresponding only with diastole, was considered severely abnormal.

These variables were classified into categorical variables as normal, mildly abnormal, or severely abnormal, as previously reported, and the hepatic portal venous pulsatility was also reported as a continuous variable from 0% to 100%. Any combination of normal or mildly abnormal scores is given a grade of 1. If the patient has one severely abnormal score, they are given a grade of 2. Two or more severely abnormal scores results in a grade of 3, reflecting severe congestion (Supplemental Table 1).

Additionally, other meaningful echocardiographic variables were also recorded during the index ultrasonographic scan including LVEF, left ventricular outflow tract area, left ventricular outflow tract velocity time integral, E/A ratio, as well as lung ultrasound profile (considered as A profile if < 3 B-lines per field or B profile, if more than 3 lines per field, using a 4-site/hemithorax scan protocol). The inclusion in the study depended on the availability of a cardiologist with training in critical care ultrasound (ie, only available during certain shifts). The results of VExUS assessment were readily available to the treating team; however, no punctual actions were recommended in any scenario.

### Study endpoints

The primary endpoint of the study was to evaluate the association between systemic venous congestion estimated by the VExUS score and the development of AKI in AHF patients. AKI was defined by the KDIGO criteria as an increase in serum creatinine by 0.3 mg/dL or more within 48 hours, an increase in serum creatinine to 1.5 times baseline or more within the last 7 days, or a urine output < 0.5 mL/kg/h for >6 hours;^4^ the presence of AKI in hospital admission was accounted as a primary endpoint. Urinary output was monitored every 6 hours during the hospital stay by the research staff; creatinine was measured daily during hospitalization to confirm peak value and at discharge to evaluate improvement.

The secondary endpoint was the composite of adverse clinical outcomes consisting of AKI, cardiogenic shock, and all-cause mortality. Cardiogenic shock was defined as hypotension (systolic blood pressure <90 mm Hg or an ongoing need for vasopressor support), end-organ hypoperfusion with an arterial lactate level of 2.0 mmol per liter or greater, and the presence of pulmonary congestion measured by either physical examination, chest X-ray, or a Pulmonary Capillary Wedge Pressure >18. Individual components of the composite endpoint were also evaluated. After hospital discharge, patients were followed by phone calls to assess clinical condition up to day 30 after the index event.

Exploratory endpoints included initiation of renal replacement therapy, mechanical circulatory support, endotracheal intubation, vasopressor initiation, inotropic initiation, and noninvasive mechanical ventilation.

### Statistical analysis

Descriptive statistics were performed for all variables, applying measures of central tendency and dispersion, or frequency and percentage, as appropriate. Bivariate analyses were performed using Student’s *t*-test or Mann-Whitney *U* test for continuous variables and chi-square or Fisher exact test for categorical variables, as appropriate.

To test the study hypotheses, Kaplan-Meier survival curves were constructed to compare the cumulative incidence of outcomes according to VExUS category, and differences were assessed using the log-rank test. Cox proportional hazards models were then used to evaluate the association between an elevated VExUS score (>1, dichotomous) and clinical endpoints, including the primary endpoint of AKI and the secondary composite endpoint of AKI, all-cause mortality, and cardiogenic shock. We then assessed the relationship between VExUS severity (graded progressively) and AKI. Finally, we examined whether individual components of the VExUS score were independently associated with AKI using logistic regression and whether the full VExUS score provided incremental predictive value compared to its individual components.

All analyses were adjusted by sex and age. Univariate logistic regression models were used to evaluate the association between clinically relevant covariates and the occurrence of AKI. From these univariate models, separate multivariable models were constructed to evaluate independent associations. Briefly, model 1 was adjusted for VExUS score and IVC diameter, and model 2 was adjusted for VExUS score, sex, age, and traditional clinical markers of congestion (jugular venous distension and peripheral edema). All statistical analyses were conducted using STATA v14.1 (StataCorp LP).

## Results

**Figure 1:**
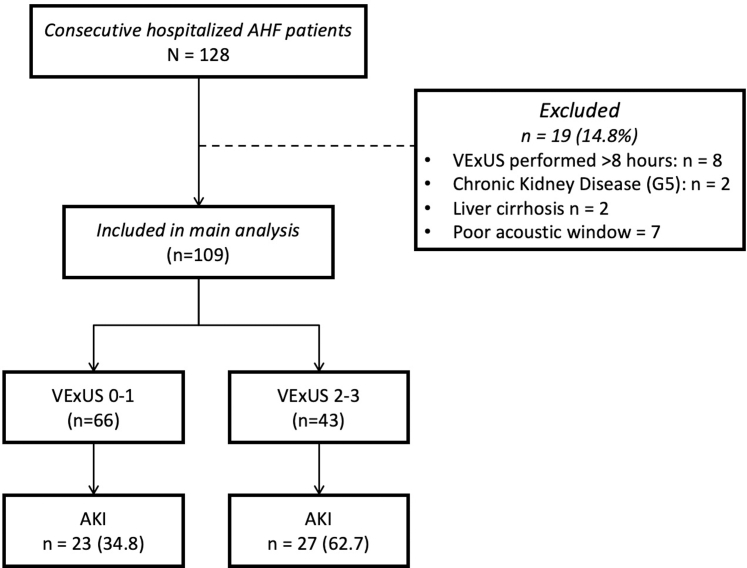
**Study Flowchart** AHF = acute heart failure; AKI = acute kidney injury; VExUS = Venous Excess Ultrasound Score.

Between July 2023 and January 2024, 128 consecutive patients with AHF were hospitalized in the study center, of whom 109 were included in the study. Figure 1 shows the study flowchart. The mean age of the study population was 58.3 (±14.23) years, and patients were predominantly male (65%). De novo HF was the most frequent diagnosis (71%), and median LVEF for the study sample was 33.5% (IQR: 19.5% to 44%). The median hospital stay was 4 (2-5) days for both groups. Table 1 shows the rest of the baseline clinical characteristics of the study population and according to the presence of severe systemic congestion (VExUS 2-3 vs 0-1).

**Table 1:** Baseline Clinical Characteristics

	Study Population *n* = 109	VExUS = 0-1 *n* = 66	VExUS 2-3 *n* = 43	*P* Value
Demographic characteristics				
Age, y	58.3 ± 14.23	59.5 ± 14.9	56.5 ± 13.0	0.281
Male, *n* (%)	71 (65)	40 (60.6)	31 (72)	0.213
Clinical presentation				
Heart rate, beats/min, median (IQR)	86 (71-100)	84 (70-100)	88 (75-101)	0.145
Systolic blood pressure, mm Hg, median (IQR)	115 (105-131)	119.5 (108-135)	110 (101-127)	0.047
Diastolic blood pressure, mm Hg, median (IQR)	75 (66-82)	73.5 (63-82)	76 (67-83)	0.801
Crackles	66 (60.5)	41 (62.1)	25 (58.1)	0.670
Peripheral edema	52 (47.7)	25 (37.8)	27 (62.7)	0.012
S3 gallop	17 (15.6)	5 (7.5)	12 (27.9)	0.004
Yugular distension	57 (52.2)	29 (43.9)	28 (65.1)	0.036
Oxygen saturation %, median (IQR)	94 (92-96)	94.5 (93-96)	94 (88-97)	0.571
Hemoglobin, g/dL, median (IQR)	14.3 (12.4-15.8)	14.7 (12.5-15.9)	13.1 (11.8-15.8)	0.108
Creatinine, median (IQR)	1.26 (0.91-2.1)	1.18 (0.86-2.1)	1.39 (0.95-2.12)	0.163
Blood urea nitrogen mg/dL median (IQR)	26.5 (18.2-43.9)	23.9 (15.7-38.9)	29 (21.9-47)	0.021
NT-proBNP, pg/mL median (IQR)	8,817.5 (2,799-20,025)	6,038 (1,972-12,054)	15,834.5 (7,609-25607)	0.0001
Acute coronary syndrome during index event				
NSTEMI/UA, *n* (%)	19 (17.4)	12 (18.1)	7 (16.2)	0.843
STEMI, *n* (%)	13 (11.9)	6 (9.0)	4 (9.3)	0.762
Comorbidities and past medical history, *n* (%)				
Diabetes mellitus, *n* (%)	45 (41)	31 (47)	14 (33)	0.131
Hypercholesterolemia, *n* (%)	11 (10)	8 (12)	3 (7)	0.389
Chronic kidney disease (KDIGO III-IV), *n* (%)	11 (10)	7 (10.6)	4 (9.3)	0.821
Heart failure, de novo, *n* (%)	77 (71)	53 (81)	24 (56)	0.002
Decompensated chronic HF, (%)	32 (29)	13 (19)	19 (44)	0.006
Myocardial infarction, *n* (%)	26 (24)	17 (26)	9 (21)	0.561
Acute heart failure hospitalization last 6 mo, *n* (%)	13 (12)	3 (5)	10 (23)	0.003
In-hospital therapies, *n* (%)				
Loop diuretics	98 (89.9)	59 (89.3)	39 (90.6)	0.650
Loop diuretics, initial bolus dosing^a^	80 (40-160)	80 (40-160)	80 (40-160)	0.423
Loop diuretics, median 24-h dose^b^	40 (20-80)	40 (20-80)	40 (20-80)	0.279
RAASi (ACEI/ARBs)	48 (44.0)	33 (50.0)	15 (34.8)	0.221
Sacubitril/Valsartan	31 (28.4)	18 (27.2)	13 (30.2)	0.846
SGLT2 inhibitors	29 (26.6)	17 (25.7)	12 (27.9)	0.627
Ventricular assist device	6 (5.5)	4 (6.0)	2 (4.6)	0.821

Baseline clinical characteristics according to the presence (or not) of severe systemic congestion (VExUS 2-3).

ACEI = angiotensin-converting enzyme inhibitor; ARB = angiotensin receptor blocker; HF = heart failure; NSTEMI, non–ST-segment elevation myocardial infarction; NT-proBNP = N-terminal pro–b-type natriuretic peptide; RAASi = renin-angiotensin-aldosterone system inhibitor; SGLT2 = sodium glucose co-transporter type 2 inhibitor; STEMI = ST-segment elevation myocardial infarction; VExUS = Venous Excess Ultrasound Score.

a Median initial IV bolus; expressed in furosemide equivalents.

b Median 24-hour dose, including the first 72 hours since hospital admission.

After VExUS assessment, patients were classified as: VExUS 0 = 42 (38.8%); VExUS 1 = 25 (23.1%); VExUS 2 = 14 (12.9%); and VExUS 3 = 27 (25.0%). Compared with patients with a VExUS 0 to 1, patients with VExUS >1 were more frequently diagnosed with chronic decompensated HF (44 vs 19%, *P* = 0.006), showed a lower systolic blood pressure on admission (110 vs 119.5, *P* = 0.047), a higher blood urea nitrogen (29 vs 23.9, *P* = 0.021), higher concentrations of NT-proBNP (15,834 pg/mL vs 6,038, *P* = <0.0001), and a higher rate of recent (<6m) HF hospitalizations (23 vs 5%, *P* = 0.003).

### VExUS and other echocardiographic parameters

VExUS and point-of-care echocardiography were performed within a median of 186 (97-385) minutes after hospital arrival. As shown in Table 2, patients in the VExUS >1 showed a higher mean inferior cava vein diameter (24.4 vs 18.2 mm, *P* < 0.001) and a higher proportion of severely abnormal hepatic vein flow (67 vs 11%, *P* < 0.001), portal vein flow (58 vs 6%, *P* < 0.001), and renal vein flow (53 vs 4%, *P* < 0.001). In addition, LVEF was lower (24 vs 36.5%, *P* = 0.02) and lung ultrasound B-lines count (6 vs 5, *P* = 0.043), E/A ratio (1.6 vs 1.2, *P* = 0.049), and volume rendering technique (2.9 vs 2.6, *P* = 0.021) were higher. Mean estimated right atrial pressure was also higher (15 vs 8, *P* < 0.001).

**Table 2:** VExUS Assessment and Visceral Congestion

	VExUS = 0-1 *n* = 66	VExUS 2-3 *n* = 43	*P* Value
Venous Excess Ultrasound Score assessment			
Inferior cava vein diameter, mm	18.2 ± 4.97	24.4 ± 3.43	<0.001
Hepatic vein flow			
Normal, *n* (%)	36 (54)	5 (12)	
Mildly abnormal, *n* (%)	23 (35)	9 (21)	
Severely abnormal, *n* (%)	7 (11)	29 (67)	
Portal vein flow			
Normal, *n* (%)	39 (60)	6 (14)	
Mildly abnormal, *n* (%)	22 (34)	12 (28)	
Severely abnormal, *n* (%)	4 (6)	25 (58)	
Renal vein flow			<0.001
Normal, *n* (%)	46 (71)	2 (5)	
Mildly abnormal, *n* (%)	16 (25)	18 (42)	
Severely abnormal, *n* (%)	3 (4)	23 (53)	
Selected echocardiographic parameters			
Left ventricular ejection fraction (%), median (IQR)	36.5 (23.5-46.5)	24 (15-41.75)	0.021
Left ventricular ejection fraction			0.856
>50%, (%)	17 (25.7)	11 (25.5)	
<50%, (%)	49 (74.2)	32 (74.4)	
VTI LVOT, median (IQR)	15 (10.6-18)	12.95 (9.2-17.5)	0.327
E/A ratio, median (IQR)	1.2 (0.8-2.1)	1.63 (1.06-3.22)	0.049
VRT, median (IQR)	2.63 (1.9-3.09)	2.95 (2.34-3.45)	0.021
TAPSE	17.5 ± 5.01	16.44 ± 5.04	0.144
Tricuspid S wave	10.38 ± 3.20	9.48 ± 3.40	0.083
Severe tricuspid regurgitation, (%)	5 (7.5)	5 (12)	0.391
Estimated right atrial pressure	8 (8-15)	15	<0.001
Quadrants with B-lines on lung ultrasound	5 (1-7)	6 (4-8)	0.043

Results of VExUS assessment and echocardiographic parameters according to the presence of visceral congestion.

E/A = early and late; LVOT = left ventricular outflow tract; TAPSE = tricuspid annular plane systolic excursion; VRT = volume rendering technique; VTI = velocity time integral; other abbreviation as in Table 1.

### Outcomes

A total of 42 (38.8%) patients developed AKI during hospitalization according to KDIGO criteria. Among those with AKI, 25 (50%) met criteria based on serum creatinine increase, 12 (24.0%) based on urine output, and 13 (26.0%) met both criteria. When classified by severity, 29 patients (69%) had AKI stage 1, 6 patients (15%) had stage 2, and 7 patients (16%) had stage 3.

Compared with patients without AKI, patients with AKI had a lower diastolic blood pressure on admission (71.5 vs 77.0 mm Hg; *P* = 0.035), a higher median creatinine (1.96 vs 1.03 mg/dL; *P* < 0.001), a higher blood urea nitrogen (38 vs 21 mg/dL; *P* < 0.001), higher NT-proBNP (14,995 vs 4,455 pg/mL, *P* < 0.001), higher proportion of diabetes (27 vs 18%; *P* = 0.013), pre-existing chronic kidney disease KDIGO III-IV (18 vs 3.3%; *P* = 0.012), a higher rate of recent HF hospitalizations (40 vs 18%, *P* = 0.014), and were more frequently admitted with the diagnosis of chronic decompensated HF (42 vs 19%; *P* = 0.008).

**Figure 2:**
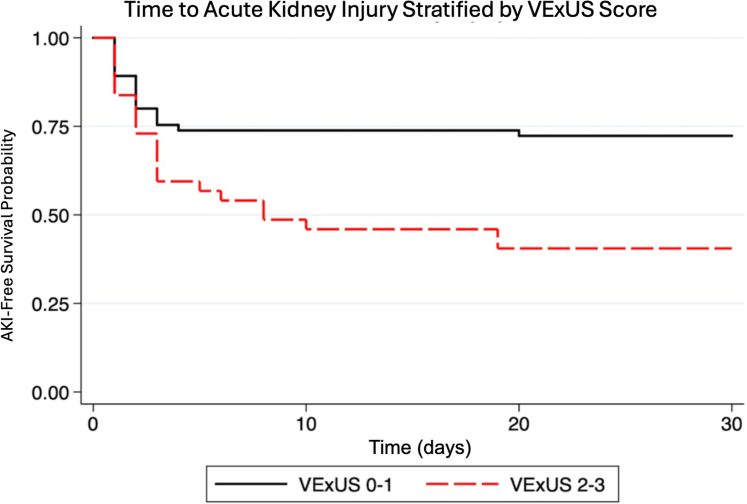
Time to Acute Kidney Injury Stratified by VExUS Score Kaplan-Meier Curve Showing a Statistically Significant Increased Risk of Acute Kidney Injury and The Composite of Acute Kidney Injury, Cardiogenic Shock, and All-Cause Mortality in Patients With Severe Systemic Congestion as Indicated by Venous Excess Ultrasound Score >1. Abbreviations as in Figure 1.

A statistically significant increased risk of AKI (HR: 2.65; 95% CI: 1.39-5.07; *P* = 0.003) and the composite of AKI, cardiogenic shock, and all-cause mortality (HR: 2.28; 95% CI: 1.21-4.26; *P* = 0.010) were found in patients showing severe systemic congestion (VExUS >1); these associations remained statistically significant after adjusting for right-sided heart failure clinical signs (peripheral edema and jugular vein ingurgitation) and echocardiographic findings (tricuspid annular plane systolic excursion, tricuspid S’ wave, diastolic right ventricle diameter) (Figure 2, Kaplan-Meier). No statistically significant differences were found in the risk of cardiogenic shock (HR: 0.64; 95% CI: 0.20-1.98; *P* = 0.445) or all-cause mortality (HR: 1.36; 95% CI: 0.28-6.60; *P* = 0.698). The associations between VExUS category at admission and other clinically relevant variables are summarized in Table 3.

**Table 3:** Main Outcomes

	VExUS 0-1 (*n* = 67)	VexUS 2-3 (*n* = 41)	*P* Value	HR (95% CI)	*P* Value
Primary outcome					
Acute kidney injury, *n* (%)	20 (29.8)	22 (53.6)	0.004	2.65 (1.39-5.07)	0.003
KDIGO stage 1, *n* (%)	12 (17.9)	17 (41.4)	0.007	2.67 (1.27-5.60)	0.009
KDIGO stage 2, *n* (%)	3 (4.4)	3 (7.3)	0.478	1.80 (0.36-8.93)	0.501
KDIGO stage 3, *n* (%)	5 (7.4)	2 (4.8)	0.199	0.66 (0.12-3.41)	0.634
Secondary outcome					
Adverse clinical outcomes composite, *n* (%)	25 (37.8)	29 (67.4)	0.003	2.28, (1.21-4.26)	0.010
30-day all-cause mortality, *n* (%)	3 (4.5)	6 (13.95)	0.074	1.36 (0.28-6.60)	0.698
Cardiogenic shock, *n* (%)	9 (13.6)	5 (11.6)	0.832	0.64 (0.20-1.98)	0.445
Exploratory endpoints					
Renal replacement therapy, *n* (%)	5 (7.5)	3 (6.9)	0.985	0.97 (0.23-4.07)	0.976
Mechanical circulatory support, *n* (%)	5 (7.5)	2 (4.65)	0.586	0.65 (0.12-3.36)	0.601
Endotracheal intubation, *n* (%)	7 (10.6)	10 (23.2)	0.058	2.44 (0.93-6.43)	0.063
Vasopressor initiation, *n* (%)	13 (19.7)	11 (25.5)	0.394	1.34 (0.60-3.0)	0.462
Inotropic initiation, *n* (%)	13 (19.7)	16 (37.2)	0.021	2.11 (1.01-4.39)	0.048
Noninvasive mechanical ventilation, *n* (%)	10 (15.1)	10 (23.2)	0.232	1.63 (0.68-3.92)	0.274

Results of clinically meaningful variables according to VExUS on admission.

Abbreviation as in Table 1.

When comparing VExUS groups, only AKI KDIGO stage 1 remained significantly associated with severe systemic congestion. Patients with VExUS >1 had a higher risk of developing KDIGO stage 1 AKI compared with those with VExUS 0 to 1 (HR: 2.67; 95% CI: 1.27-5.60; *P* = 0.009). In contrast, no statistically significant association was observed between VExUS >1 and moderate-to-severe AKI (KDIGO stages 2-3) (HR: 1.08; 95% CI: 0.35-3.31; *P* = 0.894). This latter finding should be interpreted with caution, given the limited number of patients who developed KDIGO stage 2 to 3 AKI (Table 3).

**Figure 3:**
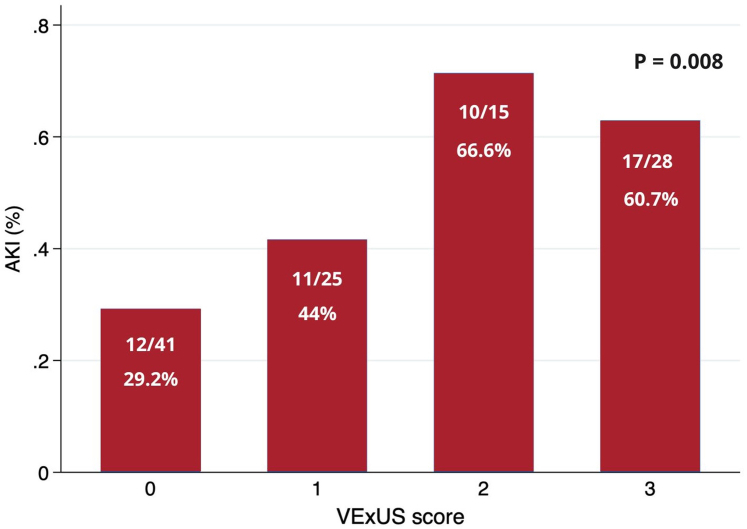
**Bar Chart** Proportion of patients with acute kidney injury according to admission and VExUS assessment in patients with acute heart failure. Abbreviations as in Figure 1.

The incidence of AKI increased with rising VExUS scores: 29.2% for VExUS 0, 44% for VExUS 1, 66.7% for VExUS 2, and 60.7% for VExUS 3 (*P* = 0.008) (Figure 3). Individual components of VExUS were associated with AKI: dilated IVC diameter (OR: 3.27; 95% CI: 1.43-4.48; *P* = 0.005), portal vein pulsatility (OR: 1.82; 95% CI: 1.12-2.95; *P* = 0.015), and intrarenal venous Doppler (OR: 1.71; 95% CI: 1.05-2.79; *P* = 0.029). The VExUS score provided incremental information compared with other physical examination or sonographic markers of congestion. In a multiple logistic regression model including IVC diameter >2 cm and VExUS >1, only VExUS score was associated with AKI (Table 4, panel A). Similarly, only VExUS score was associated with AKI in a second model including jugular venous distention and peripheral edema (Table 4, panel B).

**Table 4:** Independent Association of VExUS Score With Acute Kidney Injury After Adjustment for Other Congestion Markers

Model 1	OR
IVCd >20 mm	1.61 (0.57-4.46)
VExUS >1	**2.75 (1.01-7.85)**
Model 2	OR
JVD	2.31 (0.91-5.98)
Peripheral edema	1.34 (0.52-3.39)
VExUS >1	**2.97 (1.28-7.11)**

Association between VExUS and AKI in a multiple logistic regression model. Separate models were constructed to evaluate the incremental association of VExUS beyond isolated ultrasound-based congestion markers (IVC diameter) and beyond traditional bedside clinical signs of congestion.

IVC = inferior vena cava; IVCd = inferior vena cava diameter; JVD = jugular venous distention; other abbreviation as in Table 1.

Finally, significant covariates, including age, sex, vital signs, clinical presentation, comorbidities, and ultrasound assessments, were evaluated for their association with AKI using univariate logistic regression analysis. Variables showing a statistically significant association with AKI included pre-existing CKD (OR: 7.12; 95% CI: 1.76-48), admission NT-proBNP (OR: 9.49; 95% CI: 3.75-28.2), admission creatinine (OR: 5.52; 95% CI: 2.74-13.13), coronary artery disease (OR: 4.60; 95% CI: 1.06-32.1), and VExUS >1 (OR: 3.69; 95% CI: 1.65-8.56) among others, as summarized in Table 5.

**Table 5:** Univariate and Multivariable Analysis for the Prediction of Acute Kidney Injury

	Univariate Analysis			Multivariable Analysis		
Variables	OR	95% CI	*P* Value	OR	95% CI	*P* Value
Comorbidities						
Diabetes	2.67	1.23-5.95	*0.012*	Included in the analysis		
CKD	7.12	1.76-48	*0.023*	Included in the analysis		
Coronary heart disease	4.6	1.06-32.19	0.087	7.18	0.97-77.99	0.087
Previous heart failure	2.9	1.24-7.11	0.024	1.77	0.57-5.60	0.332
Admission diagnosis						
STEMI	0.35	0.14-0.83	0.023	0.6	0.15-2.30	0.461
Acute heart failure De novo HF	0.32	0.13-0.77	0.010	0.52	0.15-1.77	0.303
CDHF	3.16	1.36-7.7	<0.01	1.93	0.61-6.25	0.276
Admission physical exam						
Peripheral edema	2.52	1.17-5.54	0.023	1.48	0.48-4.6	0.501
Jugular venous distension	3.32	1.52-7.48	<0.01	2.83	0.82-7.32	0.068
Admission laboratory data						
NT-proBNP, pg/mL	9.49	3.75-28.22	*<0.01*	Included in the analysis		
Creatinine	5.52	2.74-13.13	*<0.01*	Included in the analysis		
Admission laboratory data						
LVEF <50%	3.44	1.22-11.31	0.031	2.49	0.59-13.53	0.256
LUS score >4	2.82	1.18-7.06	0.020	**3.92**	**1.11-16.90**	**0.042**
IVC >20 mm	2.95	1.34-6.74	<0.01	**3.87**	**1.25-13.75**	**0.039**
Abnormal portal vein Doppler	3.07	1.39-7.03	<0.01	2.9	0.98-9.36	0.067
VExUS >1	3.69	1.65-8.56	<0.01	**4.6**	**1.47-16.04**	**0.010**

Univariate logistic regression analysis evaluating the association between significant covariates and AKI. Outcome of interest: Acute kidney injury.

CDHF = chronic decompensated heart failure; CKD = chronic kidney disease; LUS = lung ultrasound; LVEF = left ventricular ejection fraction; other abbreviations as in Tables 1 and 4.

In multivariable logistic regression analysis, adjusted for diabetes, CKD, admission creatinine, and log-transformed NT-proBNP, a lung ultrasound score >4 (OR: 3.92; 95% CI: 1.11-16.9) and IVC diameter >20 mm (OR: 3.87; 95% CI: 1.25-13.75) remained significant predictors of AKI. Remarkably, VExUS >1 appeared as the strongest independent predictor of AKI during hospitalization (OR: 4.60; 95% CI: 1.47-16.0) (Central Illustration).

**Central Illustration:**
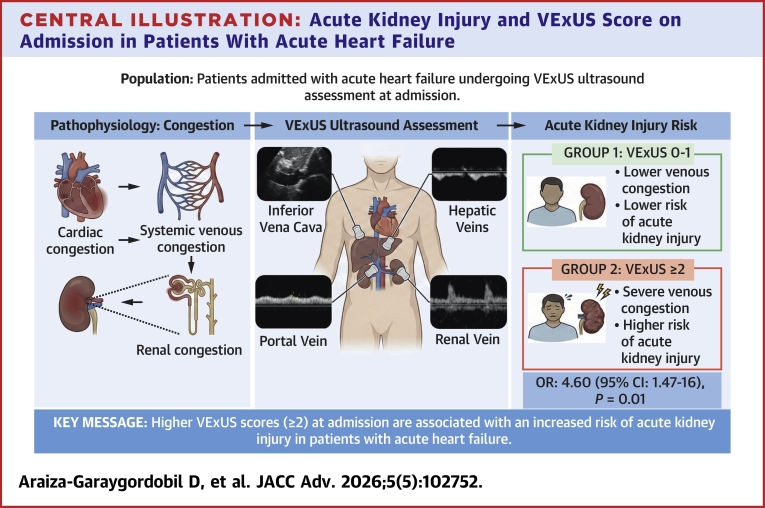
**Acute Kidney Injury and VExUS Score on Admission in Patients With Acute Heart Failure** Abbreviation as in Figure 1.

## Discussion

In the present study, the analysis of systemic venous congestion assessed on admission using VExUS revealed a significant correlation with the occurrence of AKI in patients hospitalized with AHF. To our understanding, this is the first study assessing the occurrence of AKI according to VExUS in patients with AHF.

Previously, VExUS has been identified as a predictor of AKI in several populations. Beaubien Souligny and collaborators originally described the VExUS score in 2020 and demonstrated that severe congestion assessed by VExUS was the strongest predictor of subsequent AKI development in patients undergoing cardiac surgery.^9^ This finding was later supported by Utrilla Alvarez et al (2023), who reported that the presence of grade 2 or 3 venous congestion in the postoperative period significantly increased the risk of developing AKI.^10^ More recently, we found that in patients with acute coronary syndrome, the severity of VExUS had a strong positive correlation with the incidence of AKI.^6^

It is now well accepted that right heart dysfunction and increased right-sided pressures, with consequent increased retrograde pulsatility (the so-called renal tamponade), are a cornerstone pathophysiological mechanism of AKI in AHF.^11^ However, increased right ventricular pressures are not the only pathophysiological mechanism implicated in AKI: we also know that a decreased cardiac output,^12^ the use of nephrotoxic medications,^13^ and the high prevalence of concomitant comorbidities such as diabetes and CKD are factors implicated with AKI in patients with AHF.^14^ A recent meta-analysis described that diabetes, hypertension, history of chronic kidney disease, age, NT-proBNP, eGFR <60 mL/min/1.73 m2, and blood urea nitrogen >24 mg/dL were predictor factors for developing AKI in patients with HF.^15^ Our analysis accounted for these variables and well established right ventricular failure variables such as jugular venous distention, edema, tricuspid annular plane systolic excursion, and others. Nevertheless, after multivariable regression adjusted by diabetes, CKD, serum creatinine, and NT-proBNP at admission, VExUS remained as the strongest predictor of AKI, beyond other significant ultrasonographic parameters such as LVEF, lung ultrasound score, right-sided HF parameters, and individual variables of VExUS such as IVC diameter and portal vein Doppler pulsation.

The overall association between VExUS and AKI observed in our study appears to be primarily driven by mild AKI (KDIGO stage 1). In time-to-event analyses, VExUS >1 was significantly associated with an increased risk of developing KDIGO stage 1 AKI, whereas this association was not observed for moderate-to-severe AKI (KDIGO stages 2-3). These findings are consistent with prior reports showing that stage 1 AKI accounts for the majority of AKI events among patients hospitalized with AHF. Importantly, the lack of statistical significance for KDIGO stage 2 to 3 should be interpreted cautiously, given the substantially smaller number of events in this subgroup, which limited the precision of risk estimates.

VExUS provides a more detailed assessment of the degree of venous congestion when compared to the ultrasound evaluation of IVC; this is well-documented in a recent study by Longino et al, where VExUS demonstrated an increased AUC for the detection of elevated right atrial pressure, and also for the detection of normal right atrial pressure. Furthermore, the sole evaluation of IVC may be confounded by patient-related factors (breathing patterns, obesity), technique limitations (distance from the right atrium), or anatomical variants; therefore, the addition of hepatic, portal, and renal vein pulsatility evaluation increases the detail of retrograde congestion assessment and may correct for the abovementioned limitations of the IVC (Longino et al.). Among the VExUS parameters, a proof-of-concept study suggested that portal vein Doppler may be closely related to volume status (regardless of IVC dilatation or pre-existence of severe tricuspid regurgitation), while intrarenal venous Doppler may be more related to systemic venous pressure.^16^

The potential implications of our results may include the use of VExUS as a tool to stratify the risk of AKI during hospital stay. Whether a high VExUS score on admission may lead to increased intensity of therapeutics in response (ie, increasing diuretic dosing), and whether this fact may reduce the risk of AKI is uncertain; however, several randomized controlled trials are currently being conducted (NCT06714409, NCT06397404, NCT06065163). Additionally, more information is needed to identify if serial measurements may add value to risk stratification and management strategies, as well as which cutoff points may be optimal for certain therapeutic thresholds (ie, diuretics in VExUS 1 vs VExUS >1, etc.). Something to remark regarding the present study is the higher incidence of AKI (45.8%) compared with other reports in the literature, which can vary between 10% and 40%.^17^ This may have to do with the inclusion of a critically ill population (depicted by the largely increased concentrations of NT-proBNP on admission and a low LVEF).

### Study Limitations

Our study shows limitations. First, we limited the present report to 30-day outcomes, making it difficult to assess the impact of VExUS on the long term; however, most AKI events occurred early during hospitalization; suggesting that VEXuS may be useful for short term, rather than long-term prognostication. Second, the sample size is relatively small, and the single-center nature of the study warrants the need for further, large-scale and multicenter validation of the findings, acknowledging that AKI was the main driver for a signal of increased risk in the composite endpoint (and not cardiogenic shock or all-cause mortality). Third, we do not have a complete understanding of how VExUS assessments may have affected treatment decisions in patients with AHF. It is possible that patients suffering more severe congestion were properly identified and treated aggressively, which in turn may be associated with either improved outcomes or the development of iatrogenic AKI. In this study, VExUS results were available to the treating team in all cases, but it remains uncertain whether these findings influenced management. Fourth, VExUS is an operator-dependent technique. Implementation at our institution occurred within the context of an established ultrasound program, and operators had prior experience with Doppler-based congestion assessment. Nevertheless, variability in operator training and experience may affect reproducibility and limit broader applicability of our findings. This has been explicitly acknowledged as a limitation.

## Conclusions

In patients hospitalized with AHF, VexUS is a strong predictor of AKI, beyond other hemodynamic and clinical parameters. Future studies are needed to clarify the role of VexUS assessment in patients with AHF.

## Funding support and author disclosures

Dr Arias-Mendoza has received speaking fees from AztraZeneca, 10.13039/100004319Pfizer, 10.13039/100004336Novartis, and Asofarma; and has received research fees from 10.13039/501100004191Novo Nordisk and 10.13039/100022752MSD. Dr Araiza-Garaygordobil has received speaking fees from 10.13039/100000046Abbot, Asofarma, 10.13039/100004325AstraZeneca, 10.13039/100001003Boehringer Ingelheim, 10.13039/100004336Novartis, Silanes, Servier, 10.13039/501100004191Novo Nordisk, and Lundbeck; and has received research grants from 10.13039/100004336Novartis. Dr Gopar-Nieto has received speaking fees from 10.13039/100004336Novartis and Asofarma. Dr Sierra-Lara Martinez has received speaking fees from 10.13039/100004326Bayer, 10.13039/100004336Novartis, 10.13039/501100004191Novo Nordisk, Roche, Servier, and Silanes. All other authors have reported that they have no relationships relevant to the contents of this paper to disclose.
