# Supplementary material for: Venous Excess Ultrasound Score and Acute Kidney Injury in Patients With Acute Heart Failure
Source: JACC Adv. 2026 May 27;5(5):102752. doi: 10.1016/j.jacadv.2026.102752 (PMC13221854; doi:10.1016/j.jacadv.2026.102752)
Supplement: Supplemental Table 1 [file mmc1.docx]

**Supplementary table 1**

|  | **Normal** | **Mildly Abnormal** | **Severely Abnormal** |
| --- | --- | --- | --- |
| **Hepatic vein Doppler** | **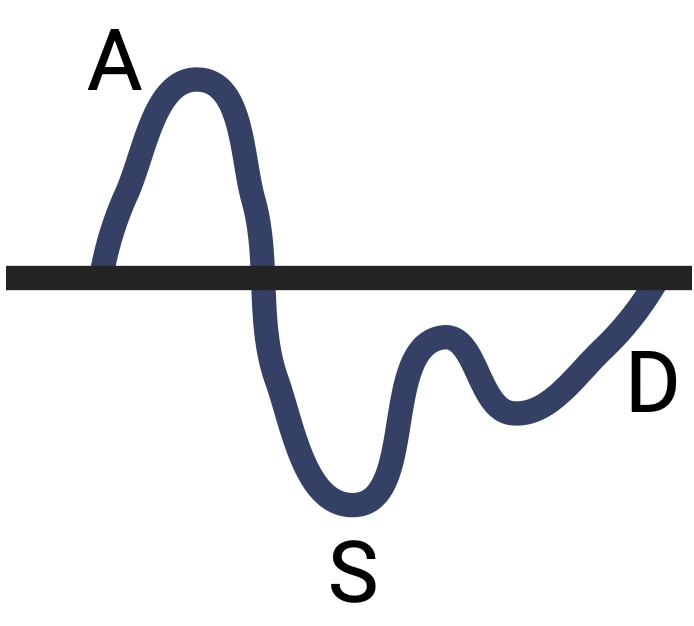** | **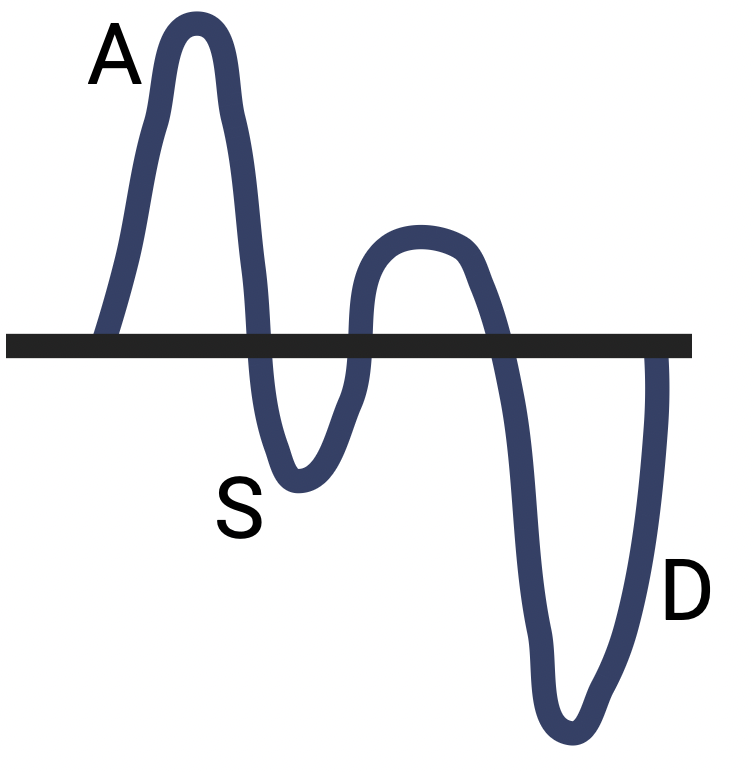** | **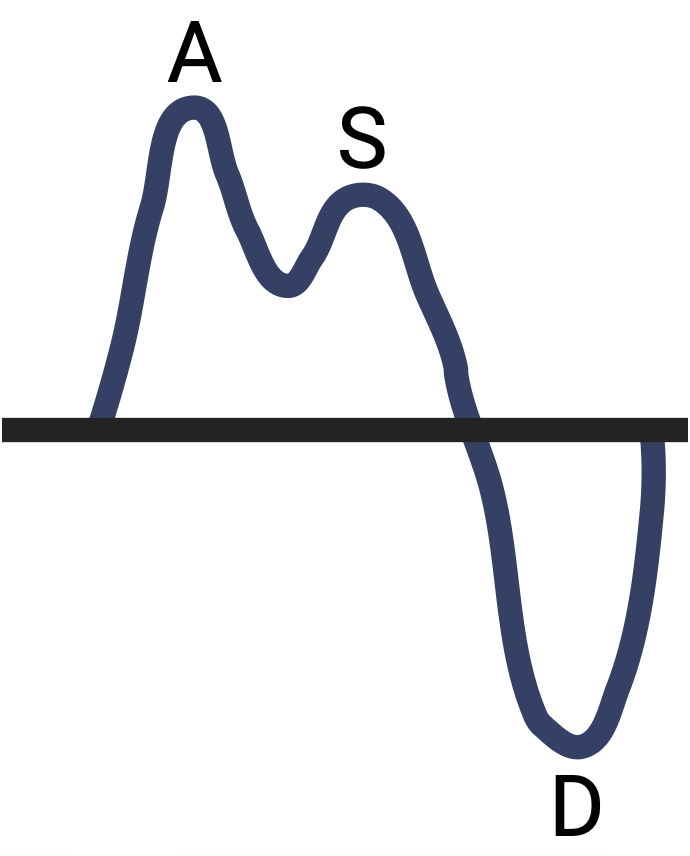** |
| **Portal vein Doppler** | 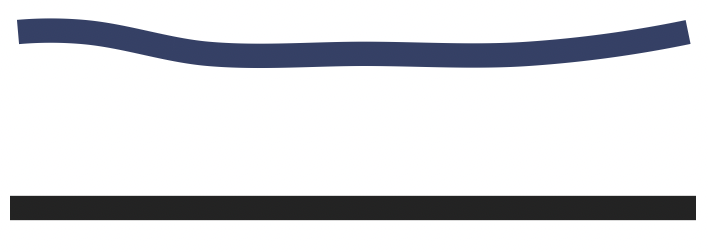  Pulsatility < 30% | 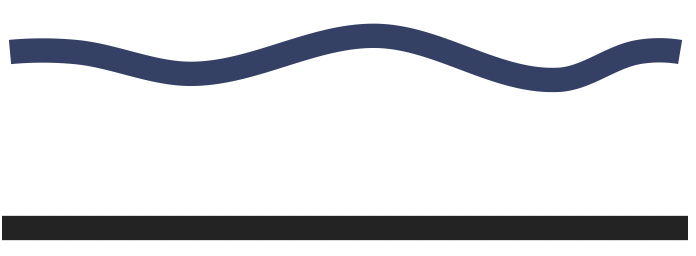  Pulsatility 30-50% | 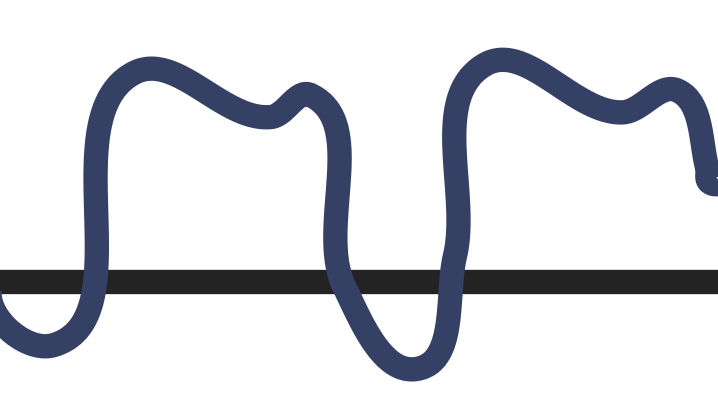  Pulsatility > 50% |
| **Intra-renal venous Doppler** | **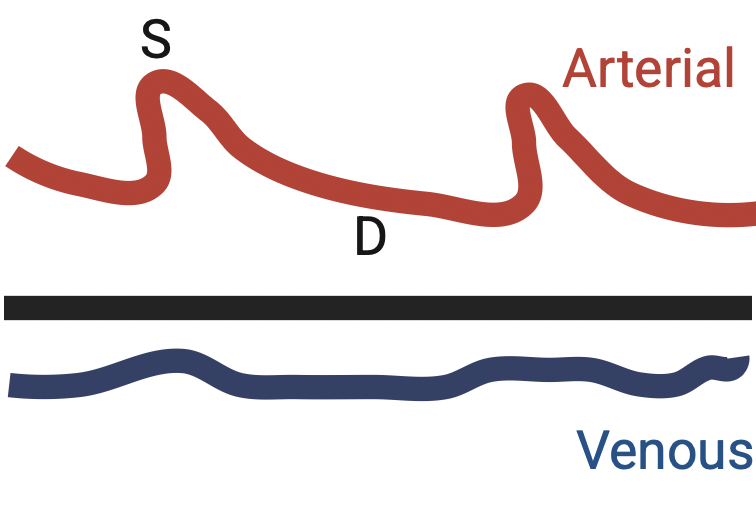**  Continuous | 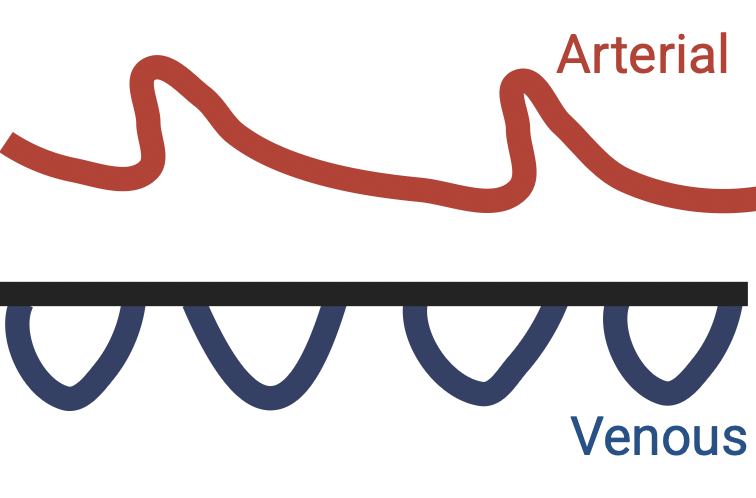  Biphasic |   Monophasic |

| **VExUS Score** | **Plethoric IVC (≥ 2.0 cm)** | **Waveform characteristics** | **Interpretation** |
| --- | --- | --- | --- |
| **Grade 0** | No | Normal | No congestion |
| **Grade 1** | Yes | Normal or mildly abnormal waveforms | Mild congestion |
| **Grade 2** | Yes | 1 severely abnormal waveform | Moderate congestion |
| **Grade 3** | Yes | 2 or more severely abnormal waveforms | Severe congestion |
